# Management of Patients with Atopic Dermatitis: The Role of Emollient Therapy

**DOI:** 10.1155/2012/836931

**Published:** 2012-09-13

**Authors:** M. Catherine Mack Correa, Judith Nebus

**Affiliations:** JOHNSON & JOHNSON Consumer Companies, Inc., 199 Grandview Road, Skillman, NJ 08558, USA

## Abstract

Atopic dermatitis is a common inflammatory skin disorder that afflicts a growing number of young children. Genetic, immune, and environmental factors interact in a complex fashion to contribute to disease expression. The compromised stratum corneum found in atopic dermatitis leads to skin barrier dysfunction, which results in aggravation of symptoms by aeroallergens, microbes, and other insults. Infants—whose immune system and epidermal barrier are still developing—display a higher frequency of atopic dermatitis. Management of patients with atopic dermatitis includes maintaining optimal skin care, avoiding allergic triggers, and routinely using emollients to maintain a hydrated stratum corneum and to improve barrier function. Flares of atopic dermatitis are often managed with courses of topical corticosteroids or calcineurin inhibitors. This paper discusses the role of emollients in the management of atopic dermatitis, with particular emphasis on infants and young children.

## 1. Introduction

**Figure 1:**
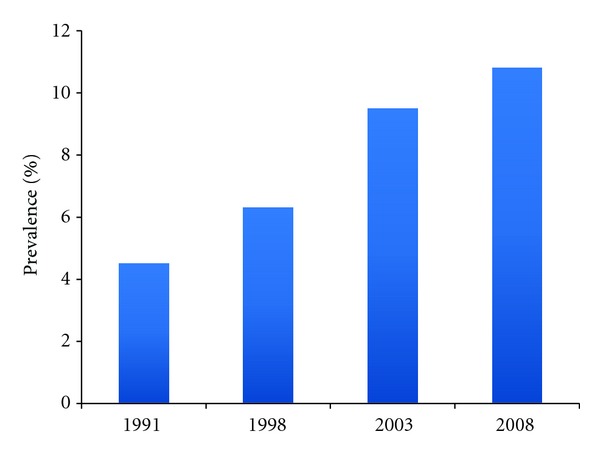
Prevalence of atopic dermatitis in Greek schoolchildren, 1991–2008 [3].

Atopic dermatitis (AD) is a skin disease characterized by inflammation, pruritus, and chronic or relapsing eczematous lesions. As one of the most common childhood skin diseases, AD afflicts approximately 17% of children in the United States [1]. Worldwide, the prevalence of symptoms for AD has generally risen, although countries with previously high rates appear to have reached a plateau [1, 2]. The increased prevalence over the last few decades is reflected in more recent data from a survey of Greek schoolchildren (Figure 1) [3]. Onset often occurs during early childhood, with 45%, 60%, and 85% of children presenting with clinical symptoms by 6 months, 1 year, and 5 years of age, respectively [4]. In the adult population, AD has an estimated lifetime prevalence of 2%–10% [4]. Although AD is a chronic disease, it resolves in about 60% of patients before adulthood.

Patients with AD frequently develop other forms of atopy. In addition to AD, food allergies are common during the first 2 years of life, with improvement during the preschool years [5]. Children with these conditions typically develop allergic rhinitis and asthma in childhood, which can persist or resolve with age [6]. The progression from AD to other forms of atopic disease is referred to as the atopic march; AD, allergic rhinitis, and asthma comprise the atopic triad. In one study, 87% of children with AD showed improvement in AD by 7 years of age, but 43% and 45% developed asthma and allergic rhinitis, respectively, by age 7 years [7]. Another study reported that rhinitis and wheezing were present in 32% and 24% of children with AD between the ages of 3 and 5 years, with mites and grass pollen identified as the most common sensitizing allergens [8]. 

Atopy—the propensity to develop hypersensitivity (overproduction of immunoglobulin E [IgE] antibodies) to allergens—is thought to underlie this progression from AD and food allergies to allergic airway diseases. There is confusion about the terms “dermatitis” and “eczema,” both of which are used interchangeably and are often associated with AD. Eczema is a broader term that is used often to describe skin diseases, including AD, allergic and irritant contact dermatitis, and seborrheic dermatitis [9]. Confusion is compounded by the medical literature, which will occasionally use the terms “AD,” “atopic eczema,” and “eczema” interchangeably. Making a clear distinction between “eczematic” skin conditions and the specific disease state of AD will help minimize confusion for patients in clinical practice. In the USA, the term “atopic” or “atopy” is used generally to describe a clinical phenotype that is associated with AD. Although “atopy” and “AD” are used interchangeably, Flohr et al. demonstrated in a systematic review that up to two-thirds of individuals with AD are not atopic (determined by IgE sensitization) [10]. These findings imply that use of the term “AD” is not accurate [10]. 

Differentiating AD from other forms of eczema is the first step in receiving a proper diagnosis. The presence of at least three major and three minor symptoms is necessary for an accurate diagnosis of AD. Major symptoms include a history of chronic or relapsing dermatitis, personal or family history of atopy, pruritus, and typical lesional morphology and distribution [11]. Whereas papules, lichenification, and excoriations characterize chronic AD, intensely pruritic erythematous papulovesicular lesions with excoriation and serous exudate characterize acute lesions in infants and young children [12]. AD rashes typically appear on the face, neck, and extensor surfaces in infants and young children, whereas AD rashes and lichenification generally appear on flexural surfaces in older children or adults with chronic disease. Early age of onset, atopy, xerosis, food intolerance, elevated IgE, and facial pallor are examples of minor symptoms that are supportive of a diagnosis [11].

Complications of AD can include secondary bacterial and viral infections, ocular abnormalities, scarring, eczema herpeticum, alterations in skin pigmentation, and sleep disturbances [13]. Sleep disturbances in infants with severe AD have been associated with behavioral changes that persist into childhood [14] and may contribute to delayed growth in children with AD [13, 15].

This narrative review provides a summary of the peer-reviewed literature that discusses AD and emollients or lotions. Studies reporting data on AD and emollients that were published between 1 January 1970 and 30 March 2012 were identified by conducting comprehensive electronic searches in PubMed. The following search terms were used individually or in combination: “atopic dermatitis,” “atopic eczema,” “atopy,” “baby,” “ceramide,” “child,” “children,” “colloidal oatmeal,” “corneocyte,” “eczema,” “emollient,” “filaggrin,” “hygiene hypothesis,” “infant,” “kallikrein,” “lotion,” “neonate,” “oatmeal,” “skin surface pH,” or “stratum corneum.” Priority was given to randomized controlled trials, but clinical studies that included small groups of participants were considered for inclusion, especially if they contained data collected from infants or children. Small clinical and in vitro studies that investigated biological phenomena underlying the etiology of AD were also considered for inclusion.

## 2. Risk Factors for Atopic Dermatitis

Genetics play a major role in AD, with parental history of atopic disease associated with both the development and severity of AD in infants. Genetic screening studies have identified more than 40 genes that have a positive association with AD [16]. Of particular interest are a cluster of genes on chromosome 1q21 that are involved in regulating epidermal homeostasis. Filaggrin, which is encoded by *FLG*, is a protein involved in the formation of natural moisturizing factor (NMF) and plays a critical role in corneocyte termination and epithelial barrier function [17, 18]. Filaggrin variants have shown a strong association with early onset and severe AD [19, 20]. In addition to being the most common gene associated with AD risk, *FLG* mutations are associated with other atopic diseases, including asthma and rhinitis [17, 21]. Other genetic mutations associated with AD include polymorphisms of lymphoepithelial Kazal-type 5 serine protease inhibitor (LEKTI or SPINK5) and human kallikrein (KLK) serine protease [22]. Both SPINK5 and KLK are involved in regulating stratum corneum (SC) structure or function [22]. SPINK5, which plays a role in the terminal differentiation of keratinocytes and epithelial formation, is colocalized with KLK proteases in the SC where it inhibits KLK5 and KLK7 [23]. Mutations in the *SPINK5* gene have been associated with AD in studies of Japanese [24, 25] and Caucasian populations [26]. Evidence of an association between KLK7 gain-of-function polymorphism and AD also has been reported [27]. Further exploration of these results and the contribution of genetic variants to AD pathophysiology is warranted.

Environmental factors contribute to the expression and severity of AD. Aeroallergens (e.g., pollen, pet dander, dust mites), food allergens, hard water, and soaps and detergents have been associated with AD [18, 28]. In one study, children with AD exhibited higher levels of sensitization to allergens compared with children without skin disorders [29]. Moreover, the severity of AD was directly associated with the degree of sensitization, particularly to dust mites and cat epithelium.

Although the prevalence of food allergy in children is approximately 6%–8%, its prevalence in children with AD ranges from 33% to 63% [30]. Development of food allergy (cow's milk, hen's egg, fish, wheat, or soybean) by 3 years of age was reported in 61% of children with AD, of whom 92% progressed to develop airborne allergies [7]. Prevalence fluctuates with severity of AD and patient age, with younger children exhibiting a higher prevalence than older children, many of whom are likely to outgrow food allergies. However, food allergy predicts persistence of AD symptoms during childhood. Avoidance of known food allergens has been reported to improve symptoms, whereas exposure can exacerbate disease.

Irritants that can exacerbate disease upon direct contact include abrasive materials such as wool and products with a propensity for causing excessive dryness, such as detergents, soaps, harsh cleansers, astringents, or alcohol. In addition, fragrance and extracts may irritate skin [6]. Studies have reported an association between hard water and increased prevalence of AD [31, 32]. It has been suggested that hard water may exacerbate AD, though a causal link has not been demonstrated [33]. The role of hard water as a contributing factor in AD is thought to be due to the presence of irritants or excessive amounts of detergents that are used frequently in hard water to produce a lather [18]. 

Other reports provide insight into the interplay between genetics and exposure to environmental factors (i.e., aeroallergens) in the risk for developing AD. In one study, the hazard ratio for developing AD was 2.26 for young children with filaggrin loss-of-function variants and 11.11 for young children with the loss-of-function variant plus neonatal cat exposure [34]. However, the presence of *FLG* mutations alone is insufficient to cause AD in all cases: 40% of children with filaggrin loss-of-function variants do not develop AD and 50% of children with AD do not have* FLG *mutations [20]. Together, these results indicate that the development of AD is a complex process that involves intrinsic and extrinsic factors that remain poorly understood.

The “hygiene hypothesis” postulates that the increase in AD and other atopic diseases is associated with improved hygiene over the decades, resulting in young children having less exposure to infectious agents, endotoxins, noninfectious microbes, and other insults [35]. Such exposure is thought to be critical in priming the maturing immune system of infants and young children. In the absence of early stimulation, the immune system overreacts to interaction with harmless agents such as dander or pollen. This hypothesis is supported by studies reporting that development of atopic disease is associated with high levels of home hygiene [36] and inversely related to multiple acute respiratory infections in young children [37, 38], the presence of older siblings, and exposure to daycare [38, 39]. However, this association remains controversial [40]. In patients with AD, an allergen can initiate an immediate IgE-mediated response as well as a delayed T-cell-mediated response [30]. The interplay between the developing immune system, environment, and genetics continues to evolve; more research is needed to elucidate the mechanisms responsible for the development and onset of atopic disease.

## 3. Physiology of Lesional and Nonlesional Skin in Atopic Dermatitis

Epidermal barrier function principally falls to the SC as the outermost skin layer. This layer has many functions, including regulating permeability and retaining moisture; protecting against ultraviolet irradiation and microorganisms; relaying mechanical and sensory signals [41]. The SC is composed of corneocytes surrounded by a continuous phase of lipids. The intercellular lipids are a mixture of ceramides, cholesterol, and free fatty acids organized into tightly packed lamellar formations [18, 42]. The amount of intercellular lipids and their organization contribute to overall SC barrier function. Corneocytes consist primarily of tightly packed keratin bundles surrounded by a cross-linked protein envelope. Ceramides are covalently bound to the outer surface of the corneocyte envelope, forming a barrier to water loss. Corneocyte hydration is also maintained by the production of NMF [18], a collection of highly hygroscopic, low-molecular-weight compounds [43, 44]. The primary source of NMF within corneocytes is the breakdown of filaggrin to its component amino acids and the derivatization of two of these amino acids, glutamine to pyrrolidone carboxylic acid, and histidine to urocanic acid [44, 45]. Urea and lactate, two compounds that are produced outside of corneocytes, comprise approximately 20% of NMF [46]. Maintenance of highly organized lipid lamellae and sufficiently hydrated, tightly bound corneocytes is critical to ensuring SC integrity.

The impaired epidermal barrier function in AD is multifactorial in nature and manifests as dysfunction in both the permeability and antimicrobial barriers of the SC. Transepidermal water loss (TEWL) has been shown to be higher than normal in skin with AD that lacks overt clinical manifestations of disease [47, 48], which indicates that the permeability barrier is disrupted even in the absence of a lesion. Increased TEWL is reported in both the presence and absence of *FLG* mutations in patients with AD, but it is higher in AD patients with *FLG* mutations [49]. This increased water loss contributes to the characteristically dryer and rougher skin of patients with AD versus those without AD. The significantly greater increase in TEWL in filaggrin-related AD versus non-filaggrin-related AD [49] is not surprising because of the role of filaggrin in production of NMF. Thus, loss-of-function mutations in the* FLG* gene leads to reduced corneocyte hydration in the SC [49, 50]. However, additional pathways also contribute to the compromised permeability of the SC. 

**Figure 2:**
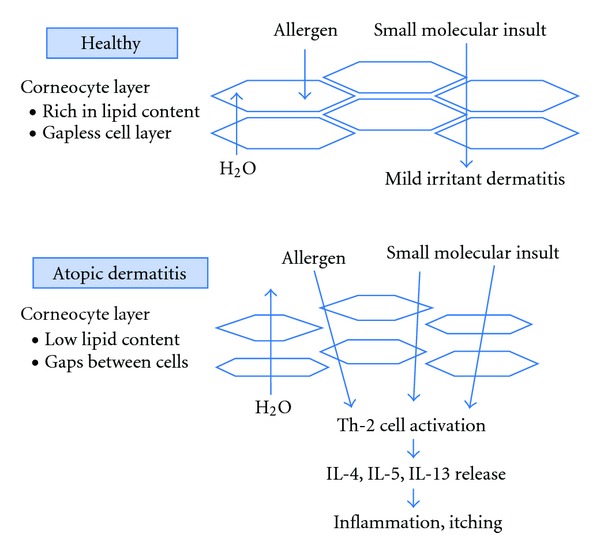
Skin of individuals with atopic dermatitis is fundamentally different compared with healthy skin.

The lipid content of the SC has been shown to be altered in AD, particularly in lesional skin. Studies have shown that in AD, the amount of ceramides in the SC is reduced [51–54], concentrations of specific ceramides species are altered [54–56], and the organization and packing of SC lipids are different than in non-AD skin [56, 57]. These changes to the SC lipid barrier contribute to increased TEWL in the skin of patients with AD [58]. Microfissures, scaling, and itching may lead to excessive scratching, which can further compromise epidermal barrier function and allow penetration of irritants and allergens [59]. Another contributing factor to the impaired permeability barrier is that corneocytes of patients with AD are significantly smaller than those in healthy individuals [60], resulting in a shorter penetration pathlength through the SC. When the barrier is compromised, allergens or microbes can penetrate the epidermal barrier, interact with antigen-presenting and immune-defector cells, and cause inflammation and itching (Figure 2). Interestingly, a fluorescence study has demonstrated that pollen penetrates the epidermal barrier via both hair follicles and the SC in healthy individuals [61]. One might extrapolate that this penetration occurs with greater ease in the skin of patients with AD.

In addition to functioning as a barrier to transport, the SC functions as an antimicrobial barrier. In AD, the antimicrobial barrier is compromised, contributing to the higher incidence of skin infections [62]. Skin surface pH, the presence of commensal microbial species, and the endogenous production of antimicrobial peptides (AMPs) are contributing factors to the antimicrobial barrier function of the SC. Skin surface pH becomes more acidic over the first several weeks of life and becomes more adult-like during the first year of life [18]. Skin surface pH in patients with AD is higher than in patients without AD [63] and is even higher in patients with flares [64]. Alterations in the skin microbiome are often observed concurrently with increased skin surface pH [65]. The microbiome of healthy skin is characterized by wide variability; commensal bacteria help to deter the growth of pathogenic bacteria (e.g., inhibition of *Staphylococcus aureus* colonization) [66]. Patients with AD demonstrate less variety in the skin microbiome, and active AD lesions are associated with particularly low bacterial diversity. Whereas *S. aureus* constitutes <5% of the microbiome in healthy individuals [65], it is the predominant microorganism in patients with AD [67] and is associated with disease severity [68]. During flares, an increase in Firmicutes (particularly *S. aureus* and *Staphylococcus epidermidis*) and a concomitant decrease in Actinobacteria (Corynebacteria, Propionibacteria) has been reported [69]. Interestingly, treatment appears to restore the diversity of the microbiome and improve the clinical measures of AD severity [69]. Although endogenous production of AMPs was once thought to be reduced during AD [70], recent evidence suggests that AMP production and expression are similar to levels observed in normal, healthy skin [71]. However, normal production of AMPs in AD may not be sufficient to counteract the increase in bacterial colonization on the skin surface.

In addition to modulating the skin microbiome, an elevated skin surface pH has been associated with delayed epidermal barrier recovery [72], as well as activation of serine proteases that lead to corneodesmosome degradation and compromised SC function [73]. A number of serine proteases are involved in desquamation, including KLK5, KLK7, and KLK14, which are localized in granular keratinocytes and the SC [18]. In the presence of a neutral or slightly alkaline pH, inactive precursors of these enzymes are cleaved into active proteases, which in turn activate other members of the cascade, leading to desquamation. Other proteases involved in corneodesmosome degradation are active in more acidic pH, including cysteine proteases (cathepsin L2, SC cathepsin-L-like enzyme) and an aspartate protease (cathepsin D) [18]. Maintenance of a skin pH gradient is necessary to regulate protease and protease inhibitor activity, thus maintaining optimal desquamation.

Most KLK proteases, particularly KLK7, exhibit increased expression in patients with AD [74]. Other proteases that contribute to skin barrier dysfunction are associated with the inflammatory response and increase with the level of severity of AD episodes. The levels of mast cell chymase (a serine kinase) were found to be similar between healthy individuals and those with AD, but significantly higher in the lesions of patients with AD [75]. Chymase is overexpressed in both lesional and nonlesional AD skin and is proposed to contribute to compromised barrier function [75]. These abnormalities contribute to a dysfunctional epidermal barrier and altered cutaneous microbiome, which makes the skin of patients with AD more prone to bacterial, fungal, and viral infection.

There are important differences in the skin of infants versus older children and adults [76]. The SC and epidermis of infants (6–24 months of age) are 30% and 20% thinner, respectively, versus adults [77]. Compared with adults, corneocytes and keratinocytes are smaller in size. SC hydration, which is more variable among infants, is generally lower than adult SC during the first month of life, yet SC hydration is greater than adult during infancy [76]. In a significant portion of patients, the developing skin barrier function during the first few years of life may be related to the prevalence of AD in infants and the resolution of the disease with age. 

## 4. Topical Options for Management of Clinical Symptoms

Treatment options for AD typically address skin barrier repair, barrier protection, or inflammatory or immunomodulatory components of disease.

### 4.1. Barrier Protection and Repair

The primary agents used as skin protectants in AD include colloidal oatmeal and petrolatum-based products. According to the US Food and Drug Administration (FDA), colloidal oatmeal has skin protectant properties and soothing effects that are indicated for the relief of itching and irritation due to eczema [78]. Colloidal oatmeal, an ingredient that is used in bath powders, cleansers, and moisturizers, contains a broad spectrum of components that provide a number of skin care benefits (Table 1). Colloidal oatmeal not only forms a protective film on the skin but also aids in the water-binding and moisture-retention properties in the SC. In addition, colloidal oatmeal also can serve as a pH buffer helping to maintain skin surface pH. There is a long-standing history of safety for colloidal oatmeal as a topical treatment to relieve itch and irritation associated with various xerotic dermatoses. Twice-daily use of colloidal oatmeal cream and use of a colloidal oatmeal cleanser for bathing of babies and young children with AD reported significant improvement in itching, dryness, roughness, and severity at 2 and 4 weeks of treatment compared with baseline [79]. A similar study performed in adults with AD reported that this regimen also significantly improved symptoms and severity of eczema [80]. After 4 weeks of using the colloidal oatmeal regimen, both studies demonstrated that an overall improvement in skin condition resulted in an improved quality of life (QoL) as measured by standardized QoL indices.

There are nearly two dozen compounds recognized by the FDA as having skin protective activity, including dimethicone (1%–30%), mineral oil (50%–100%, or 30%–35% when used with colloidal oatmeal), petrolatum (20%–100%), sodium bicarbonate, cocoa butter (50%–100%), glycerin (20%–45%), and lanolin (12.5%–50.0%) [78]. The important distinction between all these skin protectants is that only colloidal oatmeal, when used within specific levels, is allowed to make claims pertaining to skin protection and relief of minor skin irritations and itching due to eczema [78].

Emollients are moisturizers with properties that make skin soft or supple. They may contain a variety of components, including hygroscopic substances or humectants and lipids that help skin retain water and improve skin barrier function. Humectants (i.e., lactate, urea, and glycerin) are molecules with water-attracting properties that contribute to water retention within skin. Nemoto-Hasebe et al. proposed that low SC hydration in filaggrin-related AD could be related to a deficiency of water-binding filaggrin breakdown products (i.e., NMF) [49]. Given this consideration, inclusion of humectants in topical formulations may help compensate for the lower levels of SC hydration in filaggrin-related AD. Furthermore, inclusion of lipids in emollients may supplement the lipid component that is diminished in the SC of patients with AD. Ceramides, essential lipids that are derived from sphingolipids [82], are involved in barrier function. One study has shown that ceramide (and essential lipid) levels are lower in AD lesions [52].

Emollients may be formulated as lotions, creams, ointments, or bath products, most of which are available as cosmetic or over-the-counter (OTC) products. Emollient therapies are generally categorized as cosmetic moisturizers, OTC skin protectant creams, or cosmetic moisturizers and prescription barrier repair creams (BRCs). Although not all emollient products are indicated specifically for the treatment of AD, emollient therapy is recommended as a first-line treatment in multiple guidelines for AD [28, 83, 84]. Emollient therapy has been reported to improve AD symptoms and to have good tolerability in children as young as 6 months of age [85]. Cosmetics are more lightly regulated than drugs or devices; they are not subject to premarket review and approval, and manufacturers are not required to test products for their effectiveness [86]. However, many products are effective in treating or managing AD. 

Prescription and nonprescription barrier devices indicated for the treatment of AD include Atopiclair (Sinclair IS Pharma, London, United Kingdom), Eletone (Mission Pharmacal Company, San Antonio, TX, USA), EpiCeram (PuraCap Pharmaceutical LLC, South Plainfield, NJ, USA), MimyX (Stiefel Laboratories, Inc., Research Triangle Park, NC, USA), PruMyx (Prugen, Inc., Scottsdale, AZ, USA), and Neosalus Foam (Quinnova Pharmaceuticals, Inc., Newton, PA, USA). 

BRCs contain a mixture of ingredients that are reported to help alleviate inflammation and pruritus associated with AD and other forms of dermatitis, as well as repair the skin barrier. For example, EpiCeram contains a 3 : 1 : 1 ratio of ceramides, cholesterol, and free fatty acids, and it helps to manage and relieve burning and itching associated with various dermatoses, including AD. In a study of children 6 months to 18 years of age, EpiCeram and 0.05% fluticasone propionate treatment for 28 days led to a statistically significant improvement in AD severity [87].

One study analyzed the effect of an emollient and two barrier ointments on SC reservoir closure (i.e., the ability to prevent percutaneous absorption into the SC) [88]. Petrolatum, bees wax, and an oil-in-water emulsion containing waxes and surfactants were placed on test areas of skin in healthy volunteers, and a hydrophilic dye was applied to the surface of skin. Petrolatum and bees wax provided complete protection from dye penetration, but the commercial oil-in-water emulsion did not. The authors suggested that barrier ointments or creams used liberally may be useful for protecting against low-grade irritants, but they do not offer complete protection against insult penetration. This study highlights the importance of a barrier ointment or cream composition and the importance of creating an appropriately formulated emollient.

Although many emollients may be beneficial to skin barrier function, some emollients contain ingredients, such as the surfactant sodium lauryl sulfate (SLS), which can be disruptive to skin barrier function [89–92]. Aqueous Cream BP, a paraffin-based emollient that is registered with the British Pharmacopoeia, contains approximately 1% SLS. The surfactant SLS is an effective anionic surfactant that helps emulsify oils into cream formulations, but it can also be irritating and may induce an immune response in skin [93, 94]. Tsang and Guy showed that the Aqueous Cream BP caused a statistically significant increase in TEWL (with or without tape stripping) and a decrease in SC thickness on the left and right volar forearms of healthy adult volunteers [89]. Mohammed et al. showed that the Aqueous Cream BP applied twice daily for 28 days to the left and right volar forearms reduced the size and the progression of corneocyte maturation and led to increases in TEWL [90]. Protease activity increased, and the total amount of protein removed from skin via tape stripping decreased [90]. Danby et al. studied the effect of applying Aqueous Cream BP twice daily to the volar forearms of 13 adult volunteers with a previous history of AD [92]. Topical application of Aqueous Cream BP increased baseline TEWL by a statistically significant margin and led to a decrease in SC integrity [92]. Cork and Danby noted that the negative effects of Aqueous Cream BP on the skin barrier are most likely attributed to the presence of SLS (1% w/w), which disrupts the skin barrier by several mechanisms, including corneocyte swelling, keratin denaturation, and elevation of skin surface pH [91]. Despite its effect on the skin barrier, the Aqueous Cream BP is widely prescribed to individuals with eczema to relieve skin dryness [91].

### 4.2. Anti-Inflammatory and Immunomodulatory Therapies

Emollient therapy may be useful to help maintain skin barrier function and control symptoms of AD, but emollient use alone rarely leads to complete resolution of AD, especially in severe cases. Anti-inflammatory and immunomodulatory therapies may be necessary for moderate-to-severe AD until symptom resolution in skin (e.g., lesions, patches of dryness, or areas that are prone to flare). Prescription and OTC topical corticosteroids are the principal anti-inflammatory agents used in AD. Topical corticosteroids exert anti-inflammatory effects [84]. Studies of topical corticosteroids have investigated the effect of corticosteroid potency on symptom improvement in children with AD [84]. Topical corticosteroids are normally used for first-line treatment of acute exacerbations of moderate-to-severe AD [95]. The use of anti-inflammatory topical corticosteroids may lead to improvement or resolution of acute flares within a matter of days.

In a postmarketing safety review of children who used topical corticosteroids, the most common adverse effects (>10%) included local irritation, skin discoloration/depigmentation, and skin atrophy [96]. Use of lower potency compounds in children with AD is recommended to minimize the risk of adverse events and systemic effects [28, 84]. Concerns with topical corticosteroids include their potential for systemic effects, growth retardation, striae, telangiectasias, hypopigmentation, ocular effects, and skin atrophy, particularly on sensitive areas such as the face or neck [83, 97]. Despite these risks, a systematic review reported that physiologic changes and systemic complications were uncommon when the appropriate use instructions and dosing regimen of topical corticosteroids were followed [98].

Topical calcineurin inhibitors, such as tacrolimus and pimecrolimus, are options for the second-line treatment of moderate-to-severe AD in patients as young as 2 years of age [84]. Calcineurin inhibitors exert their immunomodulatory effects by inhibiting calcineurin, which in turn inhibits the activation of T-cells and cytokine expression. These effects are thought to be more selective than the effects of topical corticosteroids [41]. Topical calcineurin inhibitors have been associated with cases of malignancy, leading to a black box warning regarding risk of cancer with the use of these agents [99]. Although a causal relationship has not been demonstrated [41, 100], calcineurin inhibitors are reserved for second-line treatment only and are not recommended for children under 2 years of age [13, 84].

## 5. Maintenance of Skin Barrier

Management strategies for AD focus on maintaining the skin barrier and are recommended by medical societies worldwide [12, 28, 83, 84, 101, 102]. Use of mild, appropriately formulated emollients may provide benefits without interfering with skin barrier function. However, emollients alone may not control eczema or aspects of this skin disorder, especially in severe cases. Although emollient use alone may not be sufficient, prescription treatments (e.g., topical corticosteroids) are often considered to be less ideal for treatment of eczema in infants and young children. Given some of the unique challenges associated with topical corticosteroid treatment in young children [103], guidelines advocate for frequent and consistent use of emollients and avoidance of triggering factors as the foundation of AD management. As the underlying strategy of AD care, a more thorough discussion of optimal skin maintenance is warranted. 

### 5.1. Mild Cleansing

Bathing offers an opportunity for the cleansing and removal of excess scale, as well as improved skin hydration and increased penetration of topical therapies. However, bathing also can cause dryness and further impair the skin barrier. Bathing in lukewarm water for several minutes and using a moisturizing cleanser is recommended, as is gently patting skin dry followed by the liberal application of emollients [97]. Bathing in lukewarm water for 20 minutes followed by use of an occlusive emollient can also help provide symptomatic relief [12]. Guidelines note that addition of baking soda or colloidal oatmeal to the bath may provide an antipruritic effect [12].

Soaps are typically alkaline and can irritate the skin of patients with or without lesional AD. In one study, washing was shown to reduce the thickness of the SC and intracellular lipids in skin with AD, which suggests further impairment of epidermal barrier function [104]. In a study of individuals with and without AD, the penetration of SLS, a common ingredient used in soaps, shampoos, and bubble bath formulations, was examined *in vivo* using TEWL and tape stripping [48]. Study results showed significant penetration of SLS into the SC of uninvolved skin of patients with AD versus healthy control subjects, despite the finding that the SC thickness was the same in both groups. Additionally, in healthy skin, penetration was directly related to SC thickness, whereas SC thickness did not correlate with penetration in patients with AD. Diffusivity was twice as high in patients with AD versus controls; it was also higher in patients with active AD. This study provided further evidence that uninvolved skin in patients with AD has a defective skin barrier, which allows entry of chemicals and susceptibility to insults. These concerns are of greater importance for infants whose skin barrier and immune system has not matured fully.

Non-soap-based cleansers that support optimal skin surface pH are recommended for patients with AD [28, 102]. Guidelines recommend the use of mild synthetic detergents (syndets) with a pH of 5.5–6.0 to protect the skin's acid mantle [28]. In a 28-day study of children (≤15 years of age) with mild AD, the use of a syndet bar in place of the normal cleansing product (e.g., soap bar) resulted in less severe lesions, improved skin condition, and hydration [105]. Another study examined the effect of bathing and moisturizer combinations [106]. Results showed that the greatest level of skin hydration occurred with moisturization without a bath, whereas bathing alone reduced skin hydration, and bathing followed by moisturization provided modest hydration. It was concluded that the focus of moisturizer or emollient use should be on frequent application, regardless of the absence or presence of bathing.

Oftentimes, water contains a variety of substances that can be irritating; hard water can be especially irritating. Explanations for this association include excessive use of soap and detergent necessary to create a lather, or the presence of calcium that reacts with soap to form irritant chalk particles that enable allergen penetration and increase in cutaneous bacterial colonization [33]. The relationship between hard water and onset of AD is not understood fully. A correlation between water hardness and lifetime prevalence of eczema has been reported in several studies, but a causal relationship has not been established [31, 32, 107]. In a study that sought to address the effect of hard water, two groups of children received the same usual care, but one group also received a home water softener. Comparison of AD symptoms found no significant benefit between children receiving usual care plus the water softener versus children receiving only usual care [33].

Bathing with water alone may exacerbate clinical symptoms of AD. In a study of adults using water alone for cleansing, persistence of AD lesions was reported [108]. Even in healthy babies, bathing in water alone is not recommended due to water's drying effect on skin [109]. Babies with AD are recommended to receive regular bathing to provide skin debridement and help prevent bacterial infection. However, soap-free moisturizing liquid cleansers that do not alter skin surface pH or cause irritation or stinging are recommended [109].

### 5.2. Emollient Therapy

Guidelines recommend the consistent and liberal use of emollients and skin protectants for the prevention and maintenance of the epidermal skin barrier in patients with AD; their use may even reduce the need for topical corticosteroid use [28, 83, 84]. Emollients and skin protectants help soften the texture of skin and relieve pruritus due to excessive dryness [12]. Emollients also add a protective layer that helps aid corneocyte water retention and inhibits irritant entry [84]. A number of studies have demonstrated the benefits and safety of emollients in different age groups of patients with AD (Table 2) [79, 80, 85, 87, 110–124].

Composition of emollients can vary greatly, making one product more or less suitable for a particular individual's circumstances. Multiple emollients have been shown to improve skin barrier function, and many studies have investigated potential benefits of additional ingredients with varying mechanisms of action [126–128]. It is important to note that emollient creams, as well as cleansers, should be free of all potential allergens or irritating ingredients [12, 91].

Both prescription BRCs and OTC emollients/skin protectants can improve dry skin symptoms of AD as they protect the skin and provide irritation and pruritus relief. Emollients with ingredients such as humectants, skin conditioners, and ceramides work to moisturize the compromised dry skin barrier. Although prescription products are often assumed to be more efficacious than emollient therapy or OTC products, comparative studies provide an alternative view.

Studies have been published comparing the safety and efficacy of emollients with prescription barrier emollients. In an equivalence study, a moisturizer containing mineral oil, petrolatum, paraffin, and ceresin (Albolene, DSE Healthcare Solutions, Edison, NJ, USA) was compared with a BRC-containing glycerin, palmitoylethanolamide, pentylene glycol, olive oil, and vegetable oil (MimyX) in adults with mild-to-moderate AD [117]. Those with moderate AD also received 0.1% triamcinolone cream. All treatments were used twice daily for 4 weeks. AD parameters (erythema, desquamation, lichenification, excoriation, itching, stinging/burning, and overall severity) were assessed at baseline and weeks 1, 2, and 4. Results demonstrated that both treatments significantly improved symptoms to the same degree and with the same timing of resolution and demonstrated parity of treatments. Both treatments were well tolerated with no adverse experiences reported. Study authors noted a significant cost disparity between the therapies.

In another study, the efficacy and cost of the glycyrrhetinic acid-containing barrier cream (BRC-Gly, Atopiclair), ceramide-dominant barrier cream (BRC-Cer, EpiCeram), and OTC petroleum-based moisturizer (OTC-Pet, Aquaphor Healing Ointment, Beiersdorf Inc., Wilton, CT, USA) were compared as monotherapy for mild-to-moderate AD in children 2–17 years of age [122]. Treatments were applied three times daily for 3 weeks, with assessments performed at baseline and days 7 and 21. Assessments included 5-point Investigators Global Assessment severity scale and body surface area involved (≥1%). Improvement from baseline was noted in all three treatment groups. However, only the OTC-Pet group had statistically significant improvements in all parameters at study end. Although the OTC-Pet group had higher median percentage improvements at days 7 and 21 compared with the other treatment arms, these differences were not statistically significant. The cost of OTC skin protectant and emollient products is substantially below prescription BRCs. In the comparator study, the skin protectant was nearly 50 times more cost effective compared with the prescription BRCs [122].

### 5.3. Emollient Therapy and Reduction of Corticosteroid Usage

Because topical corticosteroids are associated with a risk of complications, including hypertrichosis, telangiectasia, skin atrophy, and stria [129], guidelines recommend that long-term use be limited [83]. To minimize adverse and systemic effects of topical corticosteroids in infants and young children with AD, appropriate potency (low or moderate, depending upon disease severity and location), duration, and localized application is recommended [84]. However, emollient monotherapy is recommended as the first approach in resolving areas of excessive dryness in very young children with AD [84].

A number of studies report a steroid-sparing effect of emollients when used in conjunction with topical corticosteroids. In a 3-week study of children with mild-to-moderate AD, once-daily hydrocortisone 2.5% cream plus an emollient (water in oil) was compared with twice-daily hydrocortisone 2.5% [130]. Skin symptoms and lesion size were significantly improved by 7 days in both treatment groups, with no significant between-group differences. These results demonstrated that the use of an emollient can be used to reduce the exposure to topical corticosteroids while providing the same degree of improvement.

In a study of infants (<12 months of age) with moderate-to-severe AD, the effect of an oat-extract containing emollient used in combination with either a moderate- or high-potency corticosteroid was examined [120]. In this 6-week study, emollient use decreased the amount of high-potency corticosteroid use by 42% (*P* < .05). The 7.5% decrease in moderate-potency steroid use was not significant. Another study in children (4–48 months of age) with moderate AD examined the effect of an oil-in-water-containing emollient on desonide 0.05% use [131]. This study found that use of topical corticosteroid every other day as adjuvant to twice-daily emollient use was as effective as monotherapy with once- or twice-daily topical corticosteroid. 

### 5.4. Controlling Clinical Symptoms of Atopic Dermatitis Through Maintenance of the Skin Barrier

Maintaining optimal hydration and addressing aspects of skin barrier dysfunction in AD may reduce the incidence of excessive dryness and irritation in AD. The fundamental approach to helping address the skin care needs of those with AD includes routinely using skin protectants and emollients, avoiding known irritants, identifying and addressing specific triggering factors, and maintaining optimal skin care [28]. A combination of approaches may be optimal for some patients.

A consensus document recommends using skin protectants/emollients at a minimum of twice daily in the presence and absence of active disease; emollients also should be applied after bathing or showering [132]. For areas of active irritation and excessive dryness, more frequent-than-normal application of skin protectants/emollients or use of an emollient with higher hydration properties can be used for management of AD [128]. There is consensus among guidelines that, regardless of which emollient is chosen, the critical aspect is that it is used consistently. Patient preference is perhaps the most important aspect of choosing an emollient, as one that is disliked will not be used. Guidelines recommend that patients with AD continuously use emollients to prevent dry skin and irritation [28, 84], with adults generally using 500–600 g per week and children using 250 g per week [128]. One set of guidelines states that the quantity of emollient used should exceed steroid use by a ratio of 10 : 1 [133]. Skin protectants and emollients should be applied generously all over the body, not just on localized areas of dry skin [84]. 

Although the primary function of emollient therapy is to keep skin hydrated and to maintain the skin barrier, other benefits of emollient therapy have also been reported. A pilot study enrolled 22 neonates who were considered at high risk for developing AD owing to family history [124]. Parents were advised to apply an oil-in-water petrolatum-based emollient at least once daily to their infant and to minimize soap exposure. By 24 months, only 15% of babies had developed AD, which occurred at a mean age of 11 months. In contrast, a systematic review reported that 30%–50% of high-risk babies developed AD by the age of 2 years [134]. The results of this pilot study indicate the need for further research in this area.

Given that emollient therapy alone is insufficient to prevent all irritation associated with eczema, other approaches to decrease the likelihood of flare recurrence have been examined. One approach may be to use a low-dose topical corticosteroid with an emollient. In one such study, patients (12–65 years of age) were maintained on a regimen of daily emollient therapy and either topical fluticasone propionate (0.05% cream or 0.005% ointment) or placebo used twice weekly in skin areas that were prone to flares [135]. Time to relapse was 16 weeks in the treatment group versus 6 weeks in the control group. The risk of relapse was 5.8 times lower and 1.9 times lower in the treatment groups for cream and ointment, respectively, compared with control groups. 

## 6. Conclusion

Atopic dermatitis is a prevalent inflammatory skin disorder characterized by intense pruritus and inflamed skin. AD can develop in very early childhood, yet resolution may occur as an infant ages. There is no known cure for AD, but the fundamentals of a daily skin care routine (e.g., use of a mild, non-soap-based cleanser followed by at least twice-daily liberal use of an emollient or OTC skin protectant) are essential for hydration and maintenance of the skin barrier. Although patients with AD may be tempted to discontinue use of emollient therapy when symptoms subside, such action is contraindicated. Consistent, frequent, and liberal use of emollients is recommended to maintain skin barrier function in patients with mild AD, even in the absence of lesions. Long-term management focuses on minimizing potential exacerbations by avoiding triggers and adhering to appropriate cleansing and moisturizing regimens. Topical corticosteroids and topical calcineurin inhibitors are used to treat acute flares for patients with moderate to severe cases of AD who do not respond to more aggressive emollient use. Safety concerns regarding topical corticosteroid use, especially in children, has led to efforts to minimize exposure. To this end, steroid-sparing approaches should be sought when severity necessitates the use of a topical corticosteroid. 

The care of patients with AD has evolved considerably over the last decade. Increased understanding of skin barrier dysfunction in AD has led to the formulation of a variety of new products. The role of prescription BRCs, OTC and cosmetic emollient formulations, and anti-inflammatory compounds provides diverse options for managing symptoms associated with AD. Elucidation of other mechanisms involved in barrier dysfunction is expected to result in new targets for therapies and may lead to revision of best practices for the management or treatment of AD. The role of emollients as the foundation of treatment, especially in infants and young children, is not likely to be challenged. The benefits of improving barrier function and hydration, coupled with steroid-sparing effects, render emollients a safe and effective option for managing patients with AD, particularly for infants and young children who have a continuously maturing epidermal barrier.

## Figures and Tables

**Table 1:** Composition and beneficial properties of colloidal oatmeal [81].

Component	Benefit
Proteins	Help maintain the skin barrier
Polysaccharides and lipids	Replenish the skin barrier
Vitamin E	Antioxidant
Saponins	Cleansing
Enzymes	Antioxidants

**Table 2:** Summary of studies of emollient use in neonates, infants, children, and adults with AD.

Study population	Treatment	Study duration	Efficacy	Safety
Neonates				

Neonates (*N* = 22) at high risk for AD [124]	Petrolatum-based emollient barrier cream (Cetaphil, Galderma Laboratories, Fort Worth, TX, USA)	At least once daily for up to 2 years	Observed cases: 15% developed AD. Intent-to-treat: 23% developed AD	No adverse events related to treatment

Infants				

Infants with moderate-to-severe AD, age <12 months (*N* = 173) [120]	Oat extract-containing emollient (Exomega, Laboratories Pierre Fabre, France)	Twice daily for 6 weeks	Significantly reduced use of high-potency topical corticosteroids and improved SCORAD index and QoL	Good/Very good tolerance in 94% of infants at study end. Two serious adverse events

Children				

Children with mild-to-moderate AD, age 2 months–6 years (*N* = 25) [79]	Occlusive colloidal oatmeal cream and colloidal oatmeal glycerin cleanser (AVEENO, JOHNSON and JOHNSON Consumer Companies, Inc., Skillman, NJ, USA)	Cream: twice daily for 4 weeks. Cleanser: all bathing	Significantly improved IGA scores, dryness, roughness, and mean itch scores at 2 and 4 weeks. Significantly improved QoL scores at 4 weeks	Well tolerated; no serious adverse events related to treatment
Children with mild-to-moderate AD, age 3 months–16 years (*N* = 65) [125]	Ceramide-dominant barrier emulsion (EpiCeram)	Twice daily for 3 weeks	Improved IGA, patient satisfaction, and QoL	No serious adverse events related to treatment
Children with AD, age 6 months–12 years (*N* = 76) [85]	Moisturizer milk (Exomega) versus control	Twice daily for 2 months	Significantly improved xerosis, pruritus, and QoL	Tolerance rated as satisfactory or excellent in 97%
Children with mild-to-moderate AD, age 6 months–12 years (*N* = 142) [114]	Glycyrrhetinic acid-based cream (Atopiclair) versus vehicle	Three times daily for 43 days	Significantly improved IGA, reduced use of rescue medication (topical corticosteroid)	No serious adverse events related to treatment
Children with moderate-to-severe AD, age 6 months–18 years (*N* = 121) [87]	Ceramide-dominant barrier emulsion (EpiCeram) versus fluticasone cream (Cutivate, PharmaDerm, Melville, NY, USA)	Twice daily for 28 days	Significantly improved SCORAD index. Comparable efficacy between treatment arms	No serious adverse events related to treatment
Children with stubborn-to-recalcitrant AD, age 1.5–12.0 years (*N* = 24) [116]	Ceramide-dominant barrier emollient (TriCeram, Osmotics Corp, Denver, CO, USA) replaced prior moisturizer. Topical tacrolimus or corticosteroid was continued	Twice daily for 12 weeks, then once daily for 9 weeks	Significantly improved SCORAD in 92% of patients by 3 weeks, 100% by 21 weeks; decreased TEWL; improved SC hydration and integrity	No serious adverse events related to treatment
Children with mild-to-moderate AD, age 2–17 years (*N* = 39) [122]	Glycyrrhetinic acid-based cream (Atopiclair) versus ceramide-based barrier cream (EpiCeram) versus petrolatum-based ointment (Aquaphor Healing Ointment, Beiersdorf Inc, Wilton, CT, USA)	Three times daily for 3 weeks	All treatment arms improved, with no significant difference between treatments. Petrolatum-based ointment had greatest improvement across assessments	Well tolerated; no serious adverse events related to treatment

Adults				

Children to adults with mild-to-moderate AD, age 2–70 years [123] (Study 1, *N* = 66; study 2, *N* = 127)	Cetaphil Restoraderm moisturizer (Galderma Laboratories, Fort Worth, TX, USA)	Study 1: Twice daily for 4 weeks; study 2: twice daily for 4 weeks as adjuvant treatment with topical steroid	Study 1: significantly decreased itching and improved hydration and QoL. Study 2: versus steroid only: significantly improved hydration, decreased EASI scores and faster onset of action	No serious adverse events related to treatment
Adolescents to adults with mild-to-moderate AD, age 12–60 years (*N* = 25) [80]	Oat-based occlusive cream and oatmeal-glycerin body wash (AVEENO)	Cream: twice daily for 8 weeks. Wash: once daily	Significantly improved: EASI and IGA scores at 2, 4, and 8 weeks; QoL at 4 and 8 weeks	Well tolerated; no serious adverse events related to treatment
Adults with mild-to-moderate AD, age >16 years (*N* = 30) [111]	Glycyrrhetinic acid-based cream (Atopiclair) versus vehicle	Three times daily for 3 weeks	Significantly improved itch and EASI scores symptoms	No serious adverse events related to treatment
Adults with mild-to-moderate AD, age 2–70 years (*N* = 2456) [119]	PEA-containing barrier (MimyX)	Twice daily for 4–6 weeks	Significantly improved symptoms versus baseline, reduced use of topical corticosteroids	No serious adverse events related to treatment
Adults with AD (*N* = 197) [121]	20% glycerin versus cream base control versus cream with 4% urea + 4% sodium chloride	Once daily for 30 days	Similar improvements in dryness	Moderate-to-severe stinging in 10% of glycerin group and 24% of urea/saline group
Adults with mild-to-moderate AD (*N* = 24) [115]	20% glycerin emollient versus placebo	Twice daily for 4 weeks	Improved SC hydration, restored epidermal barrier function (TEWL)	Not reported
Adults with allergic contact dermatitis, irritant contact dermatitis, or AD (*N* = 580) [112]	Ceramide-3 plus patented nanoparticles with or without corticosteroids	Once or twice daily until clearance (8 weeks)	Significantly improved symptoms in both treatment arms. Significantly improved pruritus, erythema, fissuring, and overall severity in combination arm	Not reported
Adults with mild-to-moderate AD (*N* = 100) [113]	5% urea moisturizer versus 10% urea lotion twice daily	Twice daily for 42 days	Similar reduction in SCORAD from baseline, no difference between products	Both products well tolerated; 5 adverse events possibly related to study treatment; 3 patients withdrew from study because of adverse events
Adults with mild-to-moderate AD (*N* = 60) [117]	Mineral oil, petrolatum, and paraffin-based moisturizer (Albolene) versus barrier cream MimyX (plus 0.1% triamcinolone cream for moderate AD)	Twice daily for 4 weeks	No difference between treatment groups in clinical efficacy	No serious adverse events related to treatment
Adults with mild-to-moderate AD (*N* = 20) [118]	Hyaluronic acid-based emollient foam (Hylatopic, Onset Therapeutics, Cumberland, RI, USA) versus ceramide-containing barrier cream (EpiCeram)	Twice daily for 4 weeks	Significantly improved symptoms at weeks 2 and 4 for foam; at week 4 for cream. Patients preferred foam	No serious adverse events related to treatment
Adults with mild-to-moderate AD (*N* = 218) [110]	Glycyrrhetinic acid-based cream (Atopiclair) versus vehicle	Three times daily for 3 weeks	Significantly improved EASI and IGA, and reduced rescue medication	No serious adverse events related to treatment

AD: atopic dermatitis; SCORAD: scoring atopic dermatitis index; QoL: quality of life; IGA: investigator global assessment; TEWL: transepidermal water loss; SC: stratum corneum; EASI: eczema area and severity index; PEA: palmitoylethanolamide.

## References

[1] Spergel JM (2010). Epidemiology of atopic dermatitis and atopic march in children. *Immunology and Allergy Clinics of North America*.

[2] Asher MI, Montefort S, Björkstén B (2006). Worldwide time trends in the prevalence of symptoms of asthma, allergic rhinoconjunctivitis, and eczema in childhood: ISAAC Phases One and Three repeat multicountry cross-sectional surveys. *The Lancet*.

[3] Anthracopoulos MB, Fouzas S, Pandiora A, Panagiotopoulou E, Liolios E, Priftis KN (2011). Prevalence trends of rhinoconjunctivitis, eczema, and atopic asthma in Greek schoolchildren: four surveys during 1991–2008. *Allergy and Asthma Proceedings*.

[4] Bieber T (2010). Atopic dermatitis. *Annals of Dermatology*.

[5] Liu AH (2006). The allergic march of childhood. *MedSci Update*.

[6] Barnetson RSC, Rogers M (2002). Childhood atopic eczema. *British Medical Journal*.

[7] Gustafsson D, Sjöberg O, Foucard T (2000). Development of allergies and asthma in infants and young children with atopic dermatitis—a prospective follow-up to 7 years age. *Allergy*.

[8] Peroni DG, Piacentini GL, Bodini A, Rigotti E, Pigozzi R, Boner AL (2008). Prevalence and risk factors for atopic dermatitis in preschool children. *British Journal of Dermatology*.

[9] Johansson SGO, Bieber T, Dahl R (2004). Revised nomenclature for allergy for global use: report of the Nomenclature Review Committee of the World Allergy Organization, October 2003. *The Journal of Allergy and Clinical Immunology*.

[10] Flohr C, Johansson SGO, Wahlgren CF, Williams H (2004). How atopic is atopic dermatitis?. *The Journal of Allergy and Clinical Immunology*.

[11] Correale CE, Walker C, Murphy L, Craig TJ (1999). Atopic dermatitis: a review of diagnosis and treatment. *American Family Physician*.

[12] Leung DYM, Nicklas RA, Li JT (2004). Disease management of atopic dermatitis: an updated practice parameter. *Annals of Allergy, Asthma and Immunology*.

[13] Carbone A, Siu A, Patel R (2010). Pediatric atopic dermatitis: a review of the medical management. *The Annals of Pharmacotherapy*.

[14] Schmitt J, Chen CM, Apfelbacher C (2011). Infant eczema, infant sleeping problems, and mental health at 10 years of age: the prospective birth cohort study LISAplus. *Allergy*.

[15] Ellison JA, Patel L, Kecojevic T, Foster PJ, David TJ, Clayton PE (2006). Pattern of growth and adiposity from infancy to adulthood in atopic dermatitis. *British Journal of Dermatology*.

[16] Barnes KC (2010). An update on the genetics of atopic dermatitis: scratching the surface in 2009. *The Journal of Allergy and Clinical Immunology*.

[17] Palmer CNA, Irvine AD, Terron-Kwiatkowski A (2006). Common loss-of-function variants of the epidermal barrier protein filaggrin are a major predisposing factor for atopic dermatitis. *Nature Genetics*.

[18] Cork MJ, Danby SG, Vasilopoulos Y (2009). Epidermal barrier dysfunction in atopic dermatitis. *The Journal of Investigative Dermatology*.

[19] Barker JNWN, Palmer CNA, Zhao Y (2007). Null mutations in the filaggrin gene (FLG) determine major susceptibility to early-onset atopic dermatitis that persists into adulthood. *The Journal of Investigative Dermatology*.

[20] O’Regan GM, Irvine AD (2010). The role of filaggrin in the atopic diathesis. *Clinical and Experimental Allergy*.

[21] Weidinger S, O’Sullivan M, Illig T (2008). Filaggrin mutations, atopic eczema, hay fever, and asthma in children. *The Journal of Allergy and Clinical Immunology*.

[22] Elias PM, Hatano Y, Williams ML (2008). Basis for the barrier abnormality in atopic dermatitis: outside-inside-outside pathogenic mechanisms. *The Journal of Allergy and Clinical Immunology*.

[23] Deraison C, Bonnart C, Lopez F (2007). LEKTI fragments specifically inhibit KLK5, KLK7, and KLK14 and control desquamation through a pH-dependent interaction. *Molecular Biology of the Cell*.

[24] Kato A, Fukai K, Oiso N, Hosomi N, Murakami T, Ishii M (2003). Association of SPINK5 gene polymorphisms with atopic dermatitis in the Japanese population. *British Journal of Dermatology*.

[25] Nishio Y, Noguchi E, Shibasaki M (2003). Association between polymorphisms in the SPINK5 gene and atopic dermatitis in the Japanese. *Genes and Immunity*.

[26] Walley AJ, Chavanas S, Moffatt MF (2001). Gene polymorphism in Netherton and common atopic disease. *Nature Genetics*.

[27] Vasilopoulos Y, Cork MJ, Murphy R (2004). Genetic association between an AACC insertion in the 3′UTR of the stratum corneum chymotryptic enzyme gene and atopic dermatitis. *The Journal of Investigative Dermatology*.

[28] Akdis CA, Akdis M, Bieber T (2006). Diagnosis and treatment of atopic dermatitis in children and adults: European Academy of Allergology and Clinical Immunology/American Academy of Allergy, Asthma and Immunology/PRACTALL Consensus Report. *The Journal of Allergy and Clinical Immunology*.

[29] Schäfer T, Heinrich J, Wjst M, Adam H, Ring J, Wichmann HE (1999). Association between severity of atopic eczema and degree of sensitization to aeroallergens in schoolchildren. *The Journal of Allergy and Clinical Immunology*.

[30] Caubet JC, Eigenmann PA (2010). Allergic triggers in atopic dermatitis. *Immunology and Allergy Clinics of North America*.

[31] McNally NJ, Williams HC, Phillips DR (1998). Atopic eczema and domestic water hardness. *The Lancet*.

[32] Miyake Y, Yokoyama T, Yura A, Iki M, Shimizu T (2004). Ecological association of water hardness with prevalence of childhood atopic dermatitis in a Japanese urban area. *Environmental Research*.

[33] Thomas KS, Dean T, O’Leary C (2011). A randomised controlled trial of ion-exchange water softeners for the treatment of eczema in children. *PLoS Medicine*.

[34] Bisgaard H, Simpson A, Palmer CNA (2008). Gene-environment interaction in the onset of eczema in infancy: filaggrin loss-of-function mutations enhanced by neonatal cat exposure. *PLoS Medicine*.

[35] Okada H, Kuhn C, Feillet H, Bach JF (2010). The “hygiene hypothesis” for autoimmune and allergic diseases: an update. *Clinical and Experimental Immunology*.

[36] Sherriff A, Golding J (2002). Hygiene levels in a contemporary population cohort are associated with wheezing and atopic eczema in preschool infants. *Archives of Disease in Childhood*.

[37] Zutavern A, von Klot S, Gehring U, Krauss-Etschmann S, Heinrich J (2006). Pre-natal and post-natal exposure to respiratory infection and atopic diseases development: a historical cohort study. *Respiratory Research*.

[38] Matheson MC, Walters EH, Simpson JA (2009). Relevance of the hygiene hypothesis to early versus late onset allergic rhinitis. *Clinical and Experimental Allergy*.

[39] Flohr C, Yeo L (2011). Atopic dermatitis and the hygiene hypothesis revisited. *Current Problems in Dermatology*.

[40] Zutavern A, Hirsch T, Leupold W, Weiland S, Keil U, von Mutius E (2005). Atopic dermatitis, extrinsic atopic dermatitis and the hygiene hypothesis: results from a cross-sectional study. *Clinical and Experimental Allergy*.

[41] Plötz SG, Ring J (2010). What’s new in atopic eczema?. *Expert Opinion on Emerging Drugs*.

[42] Groen D, Poole DS, Gooris GS, Bouwstra JA (2011). Is an orthorhombic lateral packing and a proper lamellar organization important for the skin barrier function?. *Biochimica et Biophysica Acta*.

[43] Tabachnick J, LaBadie JH (1970). Studies on the biochemistry of epidermis. IV. The free amino acids, ammonia, urea, and pyrrolidone carboxylic acid content of conventional and germ-free albino guina pig epidermia. *The Journal of Investigative Dermatology*.

[44] Rawlings AV, Scott IR, Harding CR, Bowser PA (1994). Stratum corneum moisturization at the molecular level. *The Journal of Investigative Dermatology*.

[45] Sybert VP, Dale BA, Holbrook KA (1985). Ichthyosis vulgaris: identification of a defect in synthesis of filaggrin correlated with an absence of keratohyaline granules. *The Journal of Investigative Dermatology*.

[46] Lodén M (2003). Role of topical emollients and moisturizers in the treatment of dry skin barrier disorders. *American Journal of Clinical Dermatology*.

[47] Jakasa I, Koster ES, Calkoen F (2011). Skin barrier function in healthy subjects and patients with atopic dermatitis in relation to filaggrin loss-of-function mutations. *The Journal of Investigative Dermatology*.

[48] Jakasa I, de Jongh CM, Verberk MM, Bos JD, Kežić S (2006). Percutaneous penetration of sodium lauryl sulphate is increased in uninvolved skin of patients with atopic dermatitis compared with control subjects. *British Journal of Dermatology*.

[49] Nemoto-Hasebe I, Akiyama M, Nomura T, Sandilands A, McLean WHI, Shimizu H (2009). Clinical severity correlates with impaired barrier in filaggrin-related eczema. *The Journal of Investigative Dermatology*.

[50] Sergeant A, Campbell LE, Hull PR (2009). Heterozygous null alleles in filaggrin contribute to clinical dry skin in young adults and the elderly. *The Journal of Investigative Dermatology*.

[51] Imokawa G, Abe A, Jin K, Higaki Y, Kawashima M, Hidano A (1991). Decreased level of ceramides in stratum corneum of atopic dermatitis: an etiologic factor in atopic dry skin?. *The Journal of Investigative Dermatology*.

[52] Di Nardo A, Wertz P, Giannetti A, Seidenari S (1998). Ceramide and cholesterol composition of the skin of patients with atopic dermatitis. *Acta Dermato-Venereologica*.

[53] Yamamoto A, Serizawa S, Ito M, Sato Y (1991). Stratum corneum lipid abnormalities in atopic dermatitis. *Archives of Dermatological Research*.

[54] Jungersted JM, Scheer H, Mempel M (2010). Stratum corneum lipids, skin barrier function and filaggrin mutations in patients with atopic eczema. *Allergy*.

[55] Bleck O, Abeck D, Ring J (1999). Two ceramide subfractions detectable in Cer(AS) position by HPTLC in skin surface lipids of non-lesional skin of atopic eczema. *The Journal of Investigative Dermatology*.

[56] Janssens M, van Smeden J, Gooris GS (2011). Lamellar lipid organization and ceramide composition in the stratum corneum of patients with atopic eczema. *The Journal of Investigative Dermatology*.

[57] Pilgram GSK, Vissers DCJ, van der Meulen H (2001). Aberrant lipid organization in stratum corneum of patients with atopic dermatitis and lamellar ichthyosis. *The Journal of Investigative Dermatology*.

[58] Werner YLVA, Lindberg M (1985). Transepidermal water loss in dry and clinically normal skin in patients with atopic dermatitis. *Acta Dermato-Venereologica*.

[59] Kircik L (2010). A nonsteroidal lamellar matrix cream containing palmitoylethanolamide for the treatment of atopic dermatitis. *Journal of Drugs in Dermatology*.

[60] Kashibuchi N, Hirai Y, O’Goshi K, Tagami H (2002). Three-dimensional analyses of individual corneocytes with atomic force microscope: morphological changes related to age, location and to the pathologic skin conditions. *Skin Research and Technology*.

[61] Jacobi U, Engel K, Patzelt A, Worm M, Sterry W, Lademann J (2007). Penetration of pollen proteins into the skin. *Skin Pharmacology and Physiology*.

[62] Baker BS (2006). The role of microorganisms in atopic dermatitis. *Clinical and Experimental Immunology*.

[63] Eberlein-König B, Schäfer T, Huss-Marp J (2000). Skin surface pH, stratum corneum hydration, trans-epidermal water loss and skin roughness related to atopic eczema and skin dryness in a population of primary school children. *Acta Dermato-Venereologica*.

[64] Seidenari S, Giusti G (1995). Objective assessment of the skin of children affected by atopic dermatitis: a study of pH, capacitance and TEWL in eczematous and clinically uninvolved skin. *Acta Dermato-Venereologica*.

[65] Grice EA, Segre JA (2011). The skin microbiome. *Nature Reviews Microbiology*.

[66] Bibel DJ, Aly R, Bayles C, Strauss WG, Shinefield HR, Maibach HI (1983). Competitive adherence as a mechanism of bacterial interference. *Canadian Journal of Microbiology*.

[67] Leyden JJ, Marples RR, Kligman AM (1974). Staphylococcus aureus in the lesions of atopic dermatitis. *British Journal of Dermatology*.

[68] Guzik TJ, Bzowska M, Kasprowicz A (2005). Persistent skin colonization with Staphylococcus aureus in atopic dermatitis: relationship to clinical and immunological parameters. *Clinical and Experimental Allergy*.

[69] Kong HH, Oh J, Deming C (2012). Temporal shifts in the skin microbiome associated with disease flares and treatment in children with atopic dermatitis. *Genome Research*.

[70] Ong PY, Ohtake T, Brandt C (2002). Endogenous antimicrobial peptides and skin infections in atopic dermatitis. *The New England Journal of Medicine*.

[71] Schittek B (2011). The antimicrobial skin barrier in patients with atopic dermatitis. *Current Problems in Dermatology*.

[72] Mauro T, Grayson S, Gao WN (1998). Barrier recovery is impeded at neutral pH, independent of ionic effects: implications for extracellular lipid processing. *Archives of Dermatological Research*.

[73] Hachem JP, Man MQ, Crumrine D (2005). Sustained serine proteases activity by prolonged increase in pH leads to degradation of lipid processing enzymes and profound alterations of barrier function and stratum corneum integrity. *The Journal of Investigative Dermatology*.

[74] Komatsu N, Saijoh K, Kuk C (2007). Human tissue kallikrein expression in the stratum corneum and serum of atopic dermatitis patients. *Experimental Dermatology*.

[75] Badertscher K, Brönnimann M, Karlen S, Braathen LR, Yawalkar N (2005). Mast cell chymase is increased in chronic atopic dermatitis but not in psoriasis. *Archives of Dermatological Research*.

[76] Stamatas GN, Nikolovski J, Mack MC, Kollias N (2011). Infant skin physiology and development during the first years of life: a review of recent findings based on in vivo studies. *International Journal of Cosmetic Science*.

[77] Stamatas GN, Nikolovski J, Luedtke MA, Kollias N, Wiegand BC (2010). Infant skin microstructure assessed in vivo differs from adult skin in organization and at the cellular level. *Pediatric Dermatology*.

[78] Shuren J (2003). Skin protectant drug products for over-the-counter human use, final monograph. *Federal Register*.

[79] Nebus J, Wallo W Evaluating the tolerance and efficacy of a colloidal oatmeal cream and cleanser in infants and children (ages 2 months-6 years) with atopic dermatitis [poster P619].

[80] Nebus J, Wallo W, Nystrand G, Fowler JJ (2009). A daily oat-based skin care regimen for atopic skin. *Journal of the American Academy of Dermatology*.

[81] Colloidal oatmeal (2000). *The United States Pharmacopeia: The National Formulary*.

[82] Morita T, Kitagawa M, Suzuki M (2009). A yeast glycolipid biosurfactant, mannosylerythritol lipid, shows potential moisturizing activity toward cultured human skin cells: the recovery effect of MEL-a on the SDS-damaged human skin cells. *Journal of Oleo Science*.

[83] Hanifin JM, Cooper KD, Ho VC (2004). Guidelines of care for atopic dermatitis, developed in accordance with the American Academy of Dermatology (AAD)/American Academy of Dermatology Association, ‘Administrative Regulations for Evidence-Based Clinical Practice Guidelines’. *Journal of the American Academy of Dermatology*.

[84] National Collaborating Centre for Women’s and Children’s Health (2007). Atopic eczema in children. Management of atopic eczema in children from birth up to the age of 12 years. *NICE clinical guideline*.

[85] Giordano-Labadie F, Cambazard F, Guillet G, Combemale P, Mengeaud V (2006). Evaluation of a new moisturizer (Exomega milk) in children with atopic dermatitis. *The Journal of Dermatological Treatment*.

[86] Hyman PM, Carvajal R (2009). Drugs and other product choices. *Dermatologic Therapy*.

[87] Sugarman JL, Parish LC (2009). Efficacy of a lipid-based barrier repair formulation in moderate-to-severe pediatric atopic dermatitis.. *Journal of Drugs in Dermatology*.

[88] Teichmann A, Jacobi U, Waibler E, Sterry W, Lademann J (2006). An in vivo model to evaluate the efficacy of barrier creams on the level of skin penetration of chemicals. *Contact Dermatitis*.

[89] Tsang M, Guy RH (2010). Effect of Aqueous Cream BP on human stratum corneum in vivo. *British Journal of Dermatology*.

[90] Mohammed D, Matts PJ, Hadgraft J, Lane ME (2011). Influence of Aqueous Cream BP on corneocyte size, maturity, skin protease activity, protein content and transepidermal water loss. *British Journal of Dermatology*.

[91] Cork MJ, Danby S (2011). Aqueous cream damages the skin barrier. *British Journal of Dermatology*.

[92] Danby SG, Al-Enezi T, Sultan A, Chittock J, Kennedy K, Cork MJ (2011). The effect of aqueous cream BP on the skin barrier in volunteers with a previous history of atopic dermatitis. *British Journal of Dermatology*.

[93] Lammintausta K, Maibach HI, Wilson D (1987). Human cutaneous irritation: induced hyporeactivity. *Contact Dermatitis*.

[94] Walters RM, Fevola MJ, LiBrizzi JJ, Martin K (2008). Designing cleansers for the unique needs of baby skin. *Cosmetics & Toiletries*.

[95] Williams HC (2005). Clinical practice. Atopic dermatitis. *The New England Journal of Medicine*.

[96] Hengge UR, Ruzicka T, Schwartz RA, Cork MJ (2006). Adverse effects of topical glucocorticosteroids. *Journal of the American Academy of Dermatology*.

[97] Krakowski AC, Eichenfield LF, Dohil MA (2008). Management of atopic dermatitis in the pediatric population. *Pediatrics*.

[98] Callen J, Chamlin S, Eichenfield LF (2007). A systematic review of the safety of topical therapies for atopic dermatitis. *British Journal of Dermatology*.

[99] Kothary N Update on malignancies and infections in children. http://www.fda.gov/downloads/AdvisoryCommittees/CommitteesMeetingMaterials/PediatricAdvisoryCommittee/UCM204722.pdf>.

[100] Manthripragada A Topical calcineurin inhibitors and malignancies in pediatric patients: a literature review. http://www.fda.gov/downloads/AdvisoryCommittees/CommitteesMeetingMaterials/PediatricAdvisoryCommittee/UCM255411.pdf.

[101] Saeki H, Furue M, Furukawa F (2009). Guidelines for management of atopic dermatitis. *Journal of Dermatology*.

[102] Bershad SV (2011). In the clinic. Atopic dermatitis (eczema). *Annals of Internal Medicine*.

[103] Pariser D (2009). Topical corticosteroids and topical calcineurin inhibitors in the treatment of atopic dermatitis: focus on percutaneous absorption. *American Journal of Therapeutics*.

[104] White MI, Jenkinson DM, Lloyd DH (1987). The effect of washing on the thickness of the stratum corneum in normal and atopic individuals. *British Journal of Dermatology*.

[105] Solodkin G, Chaudhari U, Subramanyan K, Johnson AW, Yan X, Gottlieb A (2006). Benefits of mild cleansing: synthetic surfactant-based (syndet) bars for patients with atopic dermatitis. *Cutis*.

[106] Chiang C, Eichenfield LF (2009). Quantitative assessment of combination bathing and moisturizing regimens on skin hydration in atopic dermatitis. *Pediatric Dermatology*.

[107] Arnedo-Pena A, Bellido-Blasco J, Puig-Barbera J (2007). Domestic water hardness and prevalence of atopic eczema in Castellon (Spain) schoolchildren. *Salud Pública de México*.

[108] Uehara M, Takada K (1985). Use of soap in the management of atopic dermatitis. *Clinical and Experimental Dermatology*.

[109] Blume-Peytavi U, Cork MJ, Faergemann J, Szczapa J, Vanaclocha F, Gelmetti C (2009). Bathing and cleansing in newborns from day 1 to first year of life: recommendations from a European round table meeting. *Journal of the European Academy of Dermatology and Venereology*.

[110] Abramovits W, Boguniewicz M (2006). A multicenter, randomized, vehicle-controlled clinical study to examine the efficacy and safety of MAS063DP (Atopiclair) in the management of mild to moderate atopic dermatitis in adults. *Journal of Drugs in Dermatology*.

[111] Belloni G, Pinelli S, Veraldi S (2005). A randomised, double-blind, vehicle-controlled study to evaluate the efficacy and safety of MAS063D (Atopiclair), in the treatment of mild to moderate atopic dermatitis. *European Journal of Dermatology*.

[112] Berardesca E, Barbareschi M, Veraldi S, Pimpinelli N (2001). Evaluation of efficacy of a skin lipid mixture in patients with irritant contact dermatitis, allergic contact dermatitis or atopic dermatitis: A multicenter study. *Contact Dermatitis*.

[113] Bissonnette R, Maari C, Provost N (2010). A double-blind study of tolerance and efficacy of a new urea-containing moisturizer in patients with atopic dermatitis. *Journal of Cosmetic Dermatology*.

[114] Boguniewicz M, Zeichner JA, Eichenfield LF (2008). MAS063DP is effective monotherapy for mild to moderate atopic dermatitis in infants and children: a multicenter, randomized, vehicle-controlled study. *The Journal of Pediatrics*.

[115] Breternitz M, Kowatzki D, Langenauer M, Elsner P, Fluhr JW (2008). Placebo-controlled, double-blind, randomized, prospective study of a glycerol-based emollient on eczematous skin in atopic dermatitis: biophysical and clinical evaluation. *Skin Pharmacology and Physiology*.

[116] Chamlin SL, Kao J, Frieden IJ (2002). Ceramide-dominant barrier repair lipids alleviate childhood atopic dermatitis: changes in barrier function provide a sensitive indicator of disease activity. *Journal of the American Academy of Dermatology*.

[117] Draelos ZD (2009). An evaluation of prescription device moisturizers. *Journal of Cosmetic Dermatology*.

[118] Draelos ZD (2011). A clinical evaluation of the comparable efficacy of hyaluronic acid-based foam and ceramide-containing emulsion cream in the treatment of mild-to-moderate atopic dermatitis. *Journal of Cosmetic Dermatology*.

[119] Eberlein B, Eicke C, Reinhardt HW, Ring J (2008). Adjuvant treatment of atopic eczema: assessment of an emollient containing N-palmitoylethanolamine (ATOPA study). *Journal of the European Academy of Dermatology and Venereology*.

[120] Grimalt R, Mengeaud V, Cambazard F (2007). The steroid-sparing effect of an emollient therapy in infants with atopic dermatitis: a randomized controlled study. *Dermatology*.

[121] Lodén M, Andersson AC, Anderson C (2002). A double-blind study comparing the effect of glycerin and urea on dry, eczematous skin in atopic patients. *Acta Dermato-Venereologica*.

[122] Miller DW, Koch SB, Yentzer BA (2011). An over-the-counter moisturizer is as clinically effective as, and more cost-effective than, prescription barrier creams in the treatment of children with mild-to-moderate atopic dermatitis: a randomized, controlled trial. *Journal of Drugs in Dermatology*.

[123] Simpson E, Dutronc Y (2011). A new body moisturizer increases skin hydration and improves atopic dermatitis symptoms among children and adults. *Journal of Drugs in Dermatology*.

[124] Simpson EL, Berry TM, Brown PA, Hanifin JM (2010). A pilot study of emollient therapy for the primary prevention of atopic dermatitis. *Journal of the American Academy of Dermatology*.

[125] Kircik LH, Del Rosso JQ (2011). Nonsteroidal treatment of atopic dermatitis in pediatric patients with a ceramide-dominant topical emulsion formulated with an optimized ratio of physiological lipids. *The Journal of Clinical and Aesthetic Dermatology*.

[126] Lodén M, Andersson AC, Andersson C, Frödin T, Öman H, Lindberg M (2001). Instrumental and dermatologist evaluation of the effect of glycerine and urea on dry skin in atopic dermatitis. *Skin Research and Technology*.

[127] Kuzmina N, Hagströmer L, Emtestam L (2002). Urea and sodium chloride in moisturisers for skin of the elderly—a comparative, double-blind, randomised study. *Skin Pharmacology and Applied Skin Physiology*.

[128] Kownacki S (2009). The importance of emollients in treating the increasing incidence of atopic eczema. *Nursing Times*.

[129] Furue M, Terao H, Rikihisa W (2003). Clinical dose and adverse effects of topical steroids in daily management of atopic dermatitis. *British Journal of Dermatology*.

[130] Lucky AW, Leach AD, Laskarzewski P, Wenck H (1997). Use of an emollient as a steroid-sparing agent in the treatment of mild to moderate atopic dermatitis in children. *Pediatric Dermatology*.

[131] Msika P, De Belilovsky C, Piccardi N, Chebassier N, Baudouin C, Chadoutaud B (2008). New emollient with topical corticosteroid-sparing effect in treatment of childhood atopic dermatitis: SCORAD and quality of life improvement. *Pediatric Dermatology*.

[132] Ellis C, Luger T, Abeck D (2003). International Consensus Conference on Atopic Dermatitis II (ICCAD II): clinical update and current treatment strategies. *British Journal of Dermatology*.

[133] Primary Care Dermatology Society and British Association of Dermatologists Guidelines for the management of atopic eczema. ,http://www.bad.org.uk/Portals/_Bad/Guidelines/Clinical%20Guidelines/PCDS-BAD%20Eczema%20reviewed%202010.pdf.

[134] Hoare C, Li Wan Po A, Williams H (2000). Systematic review of treatments for atopic eczema. *Health Technology Assessment*.

[135] Berth-Jones J, Damstra RJ, Golsch S (2003). Twice weekly fluticasone propionate added to emollient maintenance treatment to reduce risk of relapse in atopic dermatitis: randomised, double blind, parallel group study. *British Medical Journal*.

